# Community Health Workers as Mental Health Paraprofessionals: Protocol for a Mixed-Methods Pilot Feasibility Study

**DOI:** 10.2196/57343

**Published:** 2024-09-12

**Authors:** Sally Moyce, Cassidy Crawford

**Affiliations:** 1 Mark and Robyn Jones College of Nursing Montana State University Bozeman, MT United States; 2 College of Health and Human Development Montana State University Bozeman, MT United States

**Keywords:** behavioral activation, community health workers, implementation science, Latino, mental health provider shortage area, pilot study, evidence-based treatments

## Abstract

**Background:**

Community health workers (CHWs) are effective in delivering behavioral activation (BA), especially in low-resource settings. In an area with a lack of Spanish-speaking mental health counselors, such as southwest Montana, CHWs can provide needed care.

**Objective:**

The goal of this pilot study protocol is to test the feasibility, acceptability, and preliminary efficacy of a model of care that engages CHWs as providers of BA.

**Methods:**

We will train 2 CHWs in BA methodology. We will enroll 20 participants who screen positive for depression in a 12-week telephone intervention for BA. Preliminary efficacy will be tested in pre- and postscores of the Beck Depression Inventory and semistructured interviews. Feasibility and acceptability will be measured through participant retention and treatment adherence. The Therapeutic Alliance with Clinician Scale will be used to measure the strength of the therapeutic relationship. Descriptive statistics will measure alliances and repeated measures ANOVA will measure trends and changes in depression scores.

**Results:**

Enrollment began in October 2023. A total of 12 participants completed at least 10 BA sessions and all study measures by the time the study concluded in May 2024. In August 2024, data analysis occurred with an anticipated manuscript to be submitted for publication in October 2024.

**Conclusions:**

Results from this study will inform future studies into the implementation of an evidence-based mental health intervention in a limited resource setting for Latino people with limited English proficiency.

**International Registered Report Identifier (IRRID):**

DERR1-10.2196/57343

## Introduction

### Background

Latino people with limited English proficiency (LEP) experience persistent and preventable mental health disparities. According to the National Alliance on Mental Illness, 18% of Latino people in the United States have a mental illness, but only 33% of those received professional services (compared to 50% of non-Hispanic Whites with mental illness) [1]. In Montana and other Mountain West regions with rapidly increasing LEP populations, the mental health infrastructure has not kept pace with the growing need. Existing English-speaking providers are only able to meet 11% of the need in rural and frontier counties of Montana [2]; however, in Montana counties where the LEP population has grown by as much as 200% [3], access to culturally and linguistically congruent mental health providers is even more dire. Through 3 years of data collection using a community-engaged approach, our team found depression in 15% of the LEP population [4]. Working with a community advisory board, we found the primary mental health concern of the LEP community to be depression and a lack of Spanish-speaking providers [5]. Montana leads the nation in suicide rates [6], making access to mental health services an urgent concern.

An evidence-based approach to increasing mental health services is the use of community health workers (CHWs) who can be specially trained to deliver mental health interventions [7]. CHWs are generally from the same community as the population they work with, and while traditional models leverage their ability to provide a bridge to health and other resources, CHWs can provide evidence-based treatments (EBTs), including preventive care and early interventions in low-resource settings, capitalizing on their language and cultural skills [8]. Evidence suggests that CHW-provided EBTs produce mental health outcomes that are comparable to professionally delivered treatments [9]. In an area with a lack of Spanish-speaking mental health counselors, such as southwest Montana, CHWs can provide much-needed services. Indeed, in preliminary work by our team, we found improvements in depression scores in patients who engaged in 6 telehealth motivational interviewing sessions with a trained CHW (Montana State University, unpublished).

CHWs have a positive impact on health outcomes and the ability to address disparities in mental health treatment in a cost-effective, community-focused way. This is especially important in a low-resource setting with a lack of Spanish-speaking providers. Therefore, the goal of this study is to test the feasibility and acceptability of a model of care that engages CHWs as providers of the evidence-based mental health intervention of behavioral activation (BA).

### Previous Studies

The theoretical underpinnings of BA suggest that depression results when a person disengages from pleasurable activities and experiences a negative mood [10]. To combat this, the BA protocol helps a client identify activities that provide pleasure and increase positive feelings. Practitioners assist the client to target core problems by scheduling specific activation activities assigned as homework [11]. Persons in BA participate in activities that are enjoyable and monitor their mood while engaging in these activities to increase their awareness of how the activity influences their mood [12]. Clients learn the connections between their activities and their moods and schedule activities to help increase engagement in activities that create positive moods [13]. Clients are also taught to identify behaviors that interfere with their goals (“depressed behaviors”) and learn ways to replace these behaviors with positive ones [14]. BA allows clients to explore personal values and goals and to identify positive behaviors that are consistent with meeting those goals. Pleasurable activities are scheduled into the daily routine, and the client tracks mood changes in response to those activities. Clients are also supported in identifying persons who may help support them in accomplishing these activities [15].

Research shows BA is an effective intervention to address depression in Latino people with LEP when delivered by Spanish-speaking mental health professionals. In a randomized hybrid efficacy and effectiveness trial, Kanter and colleagues [11] saw increased engagement and retention from the intervention group receiving BA versus the treatment-as-usual group. In another efficacy study, Latino patients diagnosed with major depressive disorder experienced a greater reduction of depressive symptoms with the implementation of BA-centered treatment when compared to treatment as usual [16]. Other comparable studies found similar results when comparing BA to treatment as usual [17,18].

However, studies on the effect of BA delivered by CHWs are limited. In one study, Magidson and colleagues [19] successfully implemented BA treatment for torture survivors in Iraq with the use of CHWs to deliver the intervention. The procedure was modified to include relevant cultural elements and account for low literacy, and researchers saw improvements in depression. A total of 2 pilot studies—one with veterans and another including the target population (US Latino people)—reported efficacy and fidelity with the use of CHWs as the public-facing component of a multidisciplinary care team [20,21].

This study will address the gap in the existing science of the effect of CHW-delivered BA intervention on depression among Latino people with LEP by testing its acceptability, feasibility, and preliminary efficacy. We will also address a disparity in mental health outcomes for Latino people with LEP in southwest Montana by increasing the supply of Spanish-language mental health resources.

## Methods

### Intervention

In total, 2 bicultural/bilingual CHWs will be recruited and trained in the administration of BA through the University of Washington’s Advancing Integrated Mental Health Solutions Center. The training, specifically designed for CHWs and other patient-facing practitioners, consists of 8 hours of training over the course of 4 months and includes both web-based asynchronous work and 5 hours of web-based training sessions with the BA professional. CHWs learned the principles of BA, how to help participants identify rewarding activities and apply activity scheduling, and how to evaluate progress. The CHWs will be supervised by a nurse who has been trained in supervising CHWs for the duration of the study. As needed, a licensed professional counselor, well versed in working with the Latino population, will be available for further supervision and case management support.

The trained CHWs will deliver a 12-week program of BA over the phone. Individual 50-minute sessions will be scheduled based on participant preference and will consist of creating a plan of activation activities, self-monitoring, identifying avoidance behaviors, setting goal-oriented behaviors, and recording mood changes with activation activities [22]. During sessions, CHWs will discuss the cycle of depression and how activities influence moods. Together, CHWs and participants will identify participant goals, create “activating” activities that help work toward those goals, and develop strategies to schedule pleasurable activities. Participants will employ identified activities as homework and track their moods with a daily log. CHWs will record participant progress, homework, and changes in mood in REDCap (Vanderbilt University).

### Participants

**Figure 1:**
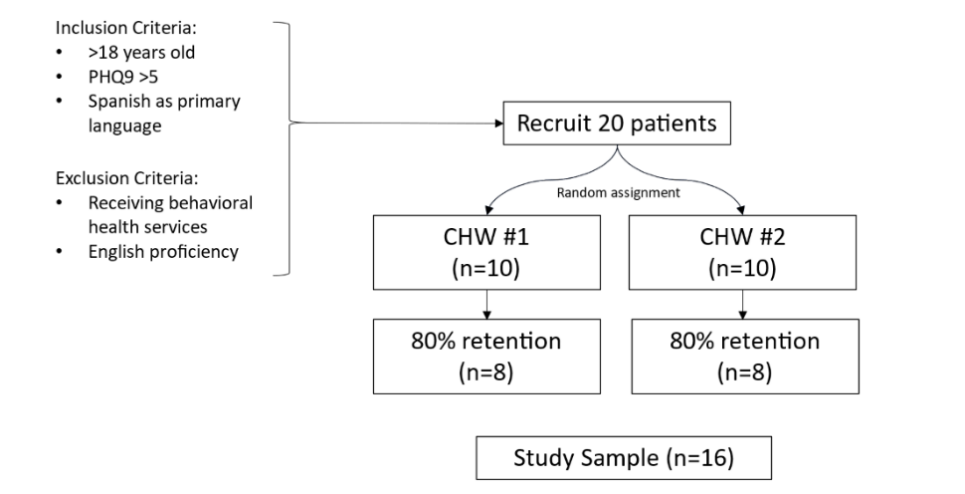
Proposed recruitment. CHW: community health worker; PHQ9: Patient Health Questionnaire-9.

We aim to recruit 20 participants for this pilot study. Recruitment will occur through a variety of community-based means: in-person community health screening events conducted by our team in Southwest Montana, referrals from the federally qualified health center (Community Health Partners) and the Gallatin City-County Health Department [4], contacts with nonprofit and service providers who work with the Latino population, and word of mouth through our community advisory boards and previous research partners. Eligible participants will be monolingual Spanish speakers with a score of at least 5 on the Patient Health Questionnaire-9 and who have the ability to participate in a phone intervention. The exclusion criteria will include current treatment by a behavioral health specialist and proficiency in English. The study enrollment goal will be 20 with an anticipated attrition rate of 20%, for a final n=16. Studies with attrition rates higher than 20% may be influenced by attrition bias [23]. Participants will be randomly assigned to one of 2 CHWs, so that each will manage a caseload of 10 participants (Figure 1). Participants will schedule their own sessions with the CHW and do not have to meet weekly. Participants who complete 10 of the 12 sessions will be considered to have completed the interventions.

### Data Collection and Analysis

We will use a mixed-methods approach to determine the acceptability, feasibility, and preliminary efficacy of the intervention. Acceptability will be measured through participant retention (number of total sessions completed of 12) and treatment adherence (percent of homework assignments completed). We will also use the Therapeutic Alliance with Clinician Scale, a 9-item Likert scale used to estimate the strength of the therapeutic relationship [24]. It has been validated in a Spanish-speaking population with an internal consistency of 0.96 [25]. Descriptive statistics will be used to determine alliance, with higher scores signaling higher estimates of alliance.

The goal of analyzing feasibility is to determine factors that contributed to the implementation of the study. For example, if the attrition rate exceeds 20%, the retention rate in the study would suggest low feasibility for participation. We will assess our methods, including participant recruitment and retention, and test the acceptability of study questionnaires. We will do this through a quantitative analysis of the counts of the number of participants who express interest versus those who enroll versus those who complete the BA sessions. One month after the study concludes, we will conduct qualitative interviews with both CHWs and a subset of participants (expected n=8). Participants who complete at least 1 session will be randomly selected by a research assistant, who will conduct interviews via telephone using a semistructured interview guide. Interviews will be audio recorded and transcribed in Spanish. We will analyze Spanish transcripts through thematic analysis [26]. Potential questions will include how participants heard about the study and why they decided to join, what aspects of the study made it easy or difficult to participate (including reasons for not completing any sessions), facilitators and barriers to completing study questionnaires, and recommendations for improvement to the study protocol. Study measures are shown in Table 1.

**Table 1:** Study implementation measures.

Outcome	Measurement	Analysis
Acceptability	Participant retention	Total sessions completed/12
	Treatment adherence	Percentage of homework assignments completed
	Therapeutic Alliance with Clinician Scale	Descriptive statistics
Efficacy	Beck Depression Inventory-II	Linear regression with repeated measures
Feasibility	Qualitative interviews	Thematic analysis

We will measure preliminary efficacy in 2 ways. First is through the semistructured interviews. We will ask participants what effect the intervention had on their general mood and if they felt the study helped improve their depression. We will also measure changes in depressive scores through the Beck Depression Inventory-II [27]. This 21-item self-report inventory is widely used to measure depression severity and has been tested in Spanish with internal consistency between 0.91 and 0.95 [28]. We will administer the Beck Depression Inventory-II at baseline, after 3 sessions, after 6 sessions, after 9 sessions, and upon completion of the BA intervention. We will use repeated measures ANOVA to estimate the trends or changes in depression scores over treatment.

### Ethical Considerations

The study was approved by the Institutional Review Board (2023-819) at Montana State University. Participants will complete a translated consent form in Spanish for participation in human studies designed by Montana State University upon enrollment in the study. They will be informed of the study objective, the logistics of the intervention, risks of participation, benefits of participation, privacy and data security protocol, and their rate of compensation. Participants in this study will be paid $200 via a prepaid VISA card upon completion of at least 10 sessions.

## Results

The study began in June 2023, and the CHWs were trained in the ethical conduct of research and BA in the fall of 2023. We began enrollment in October 2023. A total of 12 participants completed at least 10 BA sessions and all study measures by the time the study concluded in May 2024. In August 2024, data analysis occurred with an anticipated manuscript to be submitted for publication in October 2024.

## Discussion

### Anticipated Findings

We hypothesize that this study will contribute to the current science of using nonprofessionals to deliver mental health interventions to a traditionally underserved population. While CHWs have shown promise in delivering BA in many settings and are emerging as part of the evidence base for increasing access to mental health resources [8], their use in the LEP Latino population has not yet been documented. For example, a recent study by Vazquez and colleagues [29] showed that cognitive behavioral therapy delivered by CHWs among a Spanish-speaking population was feasible and acceptable. They also found reductions in depressive scores for study participants. Similarly, Steinman and colleagues [21] adapted a CHW-delivered mental health intervention that included, among other treatment modalities, BA for use with older Latino adults and found the intervention to be feasible and acceptable. In a study of an adapted BA intervention among refugees in Iraq, Bolton and colleagues found reductions in depressive scores for study participants [30]. Our project is innovative in 2 distinct ways. First, it engages a previously underserved community in the delivery of an EBT to address depression among Latino people with LEP. While BA successfully relieves depressive symptoms in Latino people with LEP, most studies use a clinical or primary care practice setting to test the intervention. Our approach is innovative and advances what we know about BA delivered by CHWs in that it engages the community itself and leverages the unique roles of CHWs to deliver an intervention outside of a traditional clinic setting via telephone. The CHWs we will employ as part of this study are currently working as CHWs in the community. They are native Spanish speakers (1 from Colombia and 1 from Peru) and work to connect community members with health services following health screening events. Second, this study will be the first of its kind to address an unmet need in Montana, where very few professional counselors have the Spanish-language skills required to develop truly therapeutic relationships with patients.

### Potential Limitations

It is conceivable that participants in our study will require a higher level of care than that which can be provided by a CHW. During our regular team meetings, if we determine that a participant requires more mental health support, they will be referred to a local federally qualified health center that provides professional mental health services, albeit through interpreters. All participants will also be counseled in the use of the 2-1-1/9-8-8 crisis line, which has services in Spanish.

Stigma impacts demand for mental health services, and stigma in the LEP Latino population [5] may be a limitation that will affect study participation. We will work closely with our community advisory boards throughout the project to address potential stigma through social media and other messaging to the community.

### Expected Impact

Successful completion of this pilot project will have a significant impact on the potential for increasing the mental health workforce for Latino people with LEP in Montana. Findings from this study—and subsequent findings from larger-scale projects—will help inform practices designed to train, employ, and reimburse CHWs. We hope to demonstrate the efficacy and feasibility of implementing an EBT approach delivered by paraprofessionals. We anticipate that a well-trained CHW workforce can help reduce mental health disparities across Montana for Latino people with LEP.

## Abbreviations

BA: behavioral activation
CHW: community health worker
EBT: evidence-based treatment
LEP: limited English proficiency

## Data Availability

The data sets that will be generated and analyzed during this study will not be publicly available due to confidentiality concerns and required compliance with informed consent protocols but will be available from the corresponding author on reasonable request.
